# Oral isotretinoin for acne vulgaris side effects on the ocular surface: Hyaluronic acid and galacto-xyloglucan as treatment for dry eye disease signs and symptoms

**DOI:** 10.3389/fmed.2022.959165

**Published:** 2022-07-22

**Authors:** María Carmen Sánchez-González, Concepción De-Hita-Cantalejo, Concepción Martínez-Lara, José-María Sánchez-González

**Affiliations:** ^1^Vision Sciences Research Group (CIVIUS), Department of Physics of Condensed Matter, Optics Area, Pharmacy School, University of Seville, Seville, Spain; ^2^Department of Nursing, University Hospital Virgen Macarena, Universidad de Sevilla, Seville, Spain; ^3^Department of Nursing, Faculty of Nursing, Physiotherapy and Podiatry, University of Seville, Seville, Spain

**Keywords:** dry eye disease, hyaluronic acid, galacto xyloglucan, isotretinoin, acne vulgaris, tear film, eyedrops

## Abstract

The purpose was to assess the efficacy of 0.4% hyaluronic acid and 0.2% galacto-xyloglucan on the subjective symptoms of dry eye disease and invasive and non-invasive tear film signs in oral isotretinoin for acne vulgaris treatment. A prospective, longitudinal, single-blind, clinical study was performed in oral isotretinoin for the acne vulgaris consumer population. Subjective dry eye disease questionnaires and invasive and non-invasive tear film assessments were reported prior to and after 6 weeks of hyaluronic acid with galacto-xyloglucan (HA-GX) treatment vs. hyaluronic acid alone (HA). Participants in the HA-GX group reported a higher decrease in the ocular surface disease index (17.01 ± 11.36 score points) compared to the variation in participants in the HA group (11.61 ± 11.18 score points). Standard patient evaluation of eye dryness also decreased more in participants in the HA-GX group (4.06 ± 5.50 score points) than in participants who received HA alone (0.70 ± 3.16). Regarding non-invasive break-up time (NIBUT), participants in the HA-GX group first NIBUT achieved an increase of 1.75 ± 1.16 s while participants in the HA-alone group demonstrated an increase of only 0.54 ± 1.01 s. The HA-GX group mean NIBUT increased by of 3.72 ± 5.69 s; however, the value for the HA-alone group was 2.19 ± 5.26 s. Hyaluronic acid in combination with galacto-xyloglucan significantly decreased limbal and bulbar conjunctival redness classification and SPEED test outcomes. The inclusion of galacto-xyloglucan also increased BUT and mean NIBUT values compared to those obtained with hyaluronic acid alone.

## Introduction

Dry eye disease (DED) is a multifactorial disease that affects the ocular surface, produces diverse symptoms and, in some cases, produces lesions that affect the anterior surface of the eye. It becomes more common with age and is more common in women than in men (1, 2). DED can be secondary to systemic diseases, especially autoimmune diseases, incomplete lid closure and infrequent blinking, excessive use of electronic devices and contact lenses, and drug administration (3). Mechanisms that can induce DED as a consequence of drug use include reduction in tear volume production, alteration in afferent nerves and reflex secretion, inflammatory effects on the glands, or direct toxicity through tears (4). Isotretinoin (13-cis-retinoic acid) is one of the active forms of vitamin A and is mainly used to treat acne and some severe skin conditions (5, 6). Systemic administration causes alteration of the function and structure of the meibomian gland and inhibits the production of lipids, generating rapid tear evaporation (7). In addition, it alters the conjunctival epithelium, affecting the morphology of goblet cells and interfering with mucin production (8). The alteration of the lipid and mucin layers causes destabilization of the tear film, generating dry eye by evaporation, increased osmolarity (9) and dry eye symptoms. This situation usually causes blepharoconjunctivitis characterized by scaling on the edge of the eyelids and eyelashes and papillary conjunctivitis (6). Recently, Andrade et al. (10) compare the ocular side effects between systemic treatment with doxycycline and low-dose oral isotretinoin in patients with moderate-to-severe papulopustular rosacea and they found that doxycycline improve meibomian gland dysfunction and ocular. Moreover, regarding the effect at the level of the tear, the use of isotretinoin is related to an increase in the bacterial flora in the conjunctiva. Egger et al. (11) and Bozkurt et al. (12) showed an increase in Staphylococcus aureus in the conjunctival sac in patients during drug treatment, which can be a source of pathogens.

Artificial tears are used in the treatment of dry eye in these patients to relieve symptoms and signs. Normally, all artificial tears have an aqueous base to which different molecules are added that improves lubrication, viscosity, osmolarity, tolerance, and residence time on the ocular surface (13). Hyaluronic acid (HA) is a polysaccharide composed of polymeric disaccharides of D-glucuronic acid and N-acetyl-D-glucosamine linked by β(1–3) and β(1–4) bonds (14). Its high capacity to retain water gives it the ability to lubricate, moisturize, and protect the external surface of the eyes. In addition, it has an antioxidant cytoprotective effect on corneal epithelial cells and high regenerative and anti-inflammatory capacity (15, 16). The use of cross-linked HA is recommended to increase the density of the molecule, delay its reabsorption and increase its effectiveness over time (16). The joint formulation of HA and other molecules that improve the effectiveness of the treatment is also recommended (17–22). Galacto-xyloglucan (GX) is a polymer formed by glucose units linked by β (1–4) bonds. Most glucose residues are linked to xylose residues by α (1–6) linkages. This xylose can be linked to galactose and fucose. This polysaccharide has a similar structure to mucin and gives it properties that mimic the natural mucosal barrier (23). Tamarind seed polysaccharide (TSP) formulations at 0.5 and 1% improve dry eye symptoms (24). The formulation of tears containing HA and GX protects the anterior surface of the eye from the effects of environmental and mechanical factors and visual stress and improves dry eye symptoms (25–27).

The purpose of our research was to assess the effect of 0.4% non-crosslinked hyaluronic acid and 0.2% galacto-xyloglucan on tear film stability and to evaluate DED signs and symptoms in subjects treated with isotretinoin.

## Materials and methods

### Design

We conducted this prospective, longitudinal, single-blind, single-center study at the Optics and Optometry cabinets of the Pharmacy School (University of Seville, Seville, Spain). This research was conducted according to the Helsinki Declaration and the Ethical Committee Board of the University of Seville.

### Subjects

All the included subjects read and sign the informed consent form. An information sheet was provided to all subjects that provided details about the study procedure. The inclusion criteria were as follows: (1) Users with active isotretinoin treatment for at least the last 2 years, (2) age between 18 and 30 years old, (3) standard patient evaluation eye disease score above 0 points, (4) invasive break-up time (BUT) under 25 s, (5) completion of all examination procedures, and (6) comprehension of the aims of this research study in its entirety and signed an informed consent form before the measurements. The exclusion criteria were as follows: (1) any previous eye surgery, (2) any systemic diseases, and (3) contact lens use.

### Materials

Non-invasive tear film analysis was performed with the Integrated Clinical Platform (ICP) Ocular Surface Analyzer (OSA) from SBM System^®^ (Orbassano, Torino, Italy). The OSA allows a full assessment of the ocular surface through a combination of dry eye disease diagnostic tests. The instrument was placed in the slit lamp tonometer hall. Within the technical data, the image resolution was 6 megapixels; the acquisition mode was multishot and movie acquisition; the focus could be manual or automatic; Placido disc and NIBUT grids were available, colored, and sensitive to infrared cameras; and the light source was infrared LED or blue and with LED. Two subjective dry eye disease questionnaires were used: the Ocular Surface Disease Index (OSDI) and the Standard Patient Evaluation of Eye Dryness (SPEED) test.

Regarding the lubricants studied, eyedrop A (hyaluronic acid and galacto-xyloglucan, HA-GX group) was 0.40% hyaluronic acid sodium salt, 0.20% galacto-xyloglucan (extracted from tamarind seed), mannitol, trisodium citrate dihydrate, citric acid monohydrate and isotonic buffered solution with a sufficient quantity for 100 milliliters (Aquoral Forte^®^, distributed by ESTEVE Pharmaceuticals^®^, Barcelona, Spain, and manufacturer by Omisan Farmaceuti^®^, Guidonia Montecelio, Italy). This eyedrop was packaged in a multidose 10-milliliter bottle. Within the control group, eyedrop B (hyaluronic acid, HA group) was 0.40% hyaluronic acid sodium salt and distilled water with ginkgo biloba, cranberry, fennel and spark asiatica, boric acid, sodium tetraborate, and sodium chloride with a sufficient quantity for 100 milliliters (Eyestil Plus^®^, SIFI, Lombardia, Italy). This eyedrop was packaged in a multidose 10-milliliter bottle.

### Examination procedure

In the first phase, subjects were included or excluded according to previously defined criteria. Subjects were randomly divided, according to simple, and computer-generated random numbers, to receive eyedrops A and B. All subjects were instructed to avoid using any lubricants or drops 1 week prior to the study. After this wash-out period was finished, subjective questionnaires and non-invasive examination with OSA, from minor to major tear film fluctuations, were performed in the following order: [1] Limbal and bulbar redness classification (LBRC) that detected the blood vessel fluidity of the conjunctiva, evaluating the redness degree with the Efron Scale (0 = normal, 1 = trace, 2 = mild, 3 = moderate and 4 = severe). [2] Lipid layer thickness (LLT) evaluation with optic interferometry, evaluating the quantity of lipids layer into 7 different pattern categories (< 15 nm—not present, ∼ 15 nm—open meshwork, ∼ 30 nm—closed meshwork, ∼ 30/80 nm—wave, ∼ 80 nm—amorphous, ∼ 80/120 nm—color fringes, ∼ 120/160 nm—abnormal color). [3] Tear meniscus height (TMH) measurement evaluates the aqueous layer and is quantified within a millimeter caliper (≤ 0.20 mm—abnormal and > 0.20 mm—normal). [4] First and mean non-invasive break-up time (FNIBUT and MNIBUT, respectively) were evaluated with a special grid cone, which evaluates the quality of the mucin layer in seconds (< 10 s—abnormal and ∼ 20 s—normal). To evaluate the meibomian glands, infrared meibography was performed with a COBRA^®^ HD fundus camera (Construzione Strumenti Oftalmici, Firenze, Italy). The degree of MGD was measured by the ImageJ method defined by Pult and Nichols (28).

In a second phase, the subjects were re-evaluated after 6 weeks with a 12-h posology to quantify the ocular surface parameters and subjective questionnaires. Finally, after a rest period of 30 min, an invasive tear film examination was performed within the fluorescein break-up time test. The temperature and humidity room examination conditions were stable during all measurements.

### Statistical analysis

Statistical analysis was performed with SPSS statistical software (version 26.0, IBM Corp., Armonk, New York, United States). Descriptive analysis was performed with the mean ± standard deviation (SD) and (range value). The normality distribution of the data was assessed with the Shapiro–Wilk test. Differences in qualitative variables were assessed with the chi-square test. The differences among the first, second and third OSA measurements were assessed with the Wilcoxon test. Differences within both eyedrop groups were analyzed with the Mann–Whitney *U*-test. The correlation study was evaluated with the Spearman Rho test. For all tests, the level of significance was established at 95% (*P*-value < 0.05). The sample size was evaluated with the GRANMO calculator (Institut Municipal d’Investigació Mèdica, Barcelona, Spain. Version 7.12). The two-sided test was used. The risk of alpha and beta was set at 5 and 20%, respectively. The estimated standard deviation (*SD*) of the differences was set at 2.06 [based on De-Hita-Cantalejo et al. (17) *SD* of the main variable], the expected minimum BUT difference was set at 2.5 s, and finally, the loss to follow-up rate was set at 0.05. This achieved a recommended sample size of 24 subjects.

## Results

**FIGURE 1:**
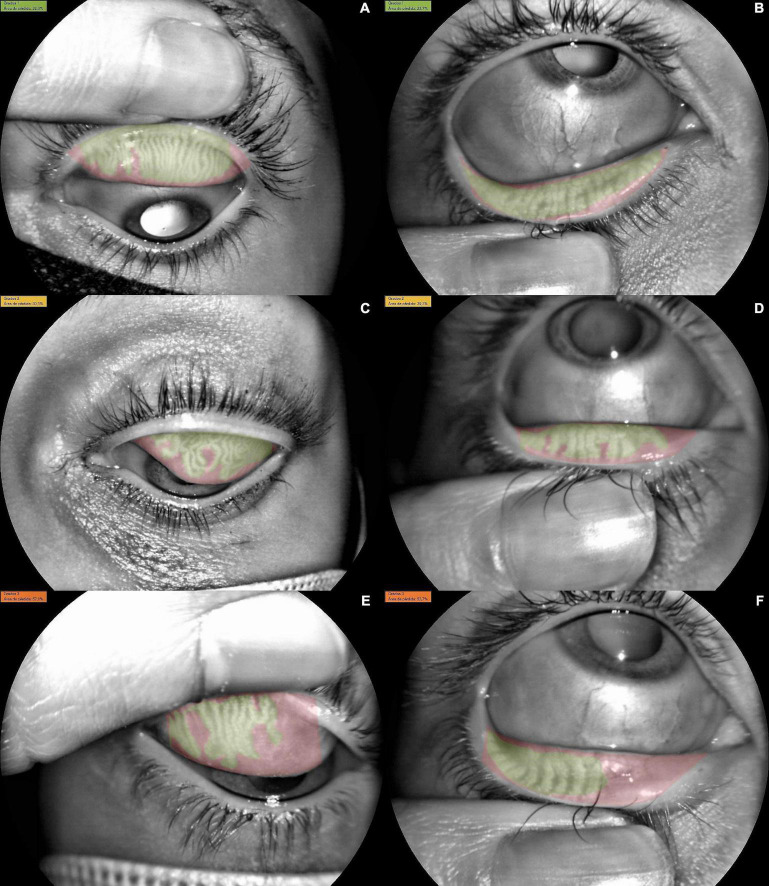
Meibomian gland dysfunction (MGD) distribution among the sample. **(A)** Superior eye lid with Grade 1 dysfunction (22.3% of MGD), **(B)** inferior eye lid with Grade 1 dysfunction (23.7% of MGD), **(C)** superior eye lid with Grade 2 dysfunction (30.1% of MGD), **(D)** inferior eye lid with Grade 2 dysfunction (39.3% of MGD). **(E)** Superior eye lid with Grade 3 dysfunction (57.1% of MGD), **(F)** inferior eye lid with Grade 3 dysfunction (53.7% of MGD).

Fifty eyes from 25 patients were included in this study. Twenty-five right eyes and 25 left eyes were included. Seven males and eighteen females were enrolled in this research. Demographic data about age, sphere refraction, cylinder refraction, axis refraction, CDVA (log MAR) and superior and inferior meibomian gland dysfunction percentages are presented in Table 1. The distribution of superior and inferior eye meibomian gland dysfunction is presented in Figure 1. Therefore, both groups were similar and comparable at the beginning of this research.

**TABLE 1:** Demographics between both eyedrop groups.

Variable	0.40% HA + 0.20% GX (*n* = 26)	0.40% HA (*n* = 24)	*P*-value
Age (years)	21.38 ± 4.18 (18.00–30.00)	20.83 ± 2.16 (18.00–24.00)	0.69
Sphere refraction (D)	–1.25 ± 2.61 (–8.50 to + 3.25)	–0.67 ± 2.50 (–4.75 to + 4.25)	0.93
Cylinder refraction (D)	–0.60 ± 0.36 (–1.50 to –0.25)	–0.83 ± 0.70 (–3.00 to –0.25)	0.39
Axis refraction (degrees)	66.37 ± 62.74 (1.00–179.00)	91.44 ± 69.59 (2.00–180.00)	0.32
CDVA (Log MAR)	0.03 ± 0.04 (0.00–0.10)	0.01 ± 0.03 (0.00–0.10)	0.15
Superior eyelid MGD (percentage)	30.58 ± 10.25 (5.40–47.50)	29.72 ± 12.12 (16.00–57.70)	0.30
Inferior eyelid MGD (percentage)	35.26 ± 11.11 (2.90–63.90)	36.55 ± 13.21 (11.00–53.70)	0.36

HA, Hyaluronic Acid; GX, Galacto Xyloglucan; D, Diopter; CDVA, Corrected Distance Visual Acuity; MGD, Meibomian Gland Dysfunction.

### Tear film non-invasive tests

Limbal and bulbar conjunctival redness, lipid layer interferometry, tear meniscus height assessment, and first and mean NIBUT differences before and after treatment are presented in Table 2 for participants in the HA-GX group and HA-alone group. Regarding differences between both groups, conjunctival redness significantly decreased 1.34 ± 0.74 grades on the Efron Scale for participants in the HA-GX group, while conjunctival redness decreased only 0.94 ± 0.77 (*P* = 0.18) for participants in the HA-alone group. Lipid layer interferometry for participants in the HA-GX group changed only 0.11 ± 1.39 degrees on the Guillon Pattern, similar to that for participants in the HA-alone group, which changed 0.12 ± 0.94 degrees on the Guillon Pattern (*P* = 0.38). The TMH for participants in the HA-GX group remained uniform, with a change of 0.009 ± 0.06 millimeters, similar to that for participants in the HA-alone group, which changed by 0.004 ± 0.03 (*P* = 0.60). Finally, regarding the first and mean NIBUT, participants in the HA-GX group achieved an increase of 0.57 ± 2.89 s in the FNIBUT; however, participants in the HA-alone group achieved a decrease of 0.64 ± 1.82 s (*P* = 0.79). In addition, the participants in HA-GX group reported an increase of 1.11 ± 5.10 s in the MNIBUT, while participants in the HA-alone group showed a decrease of 1.82 ± 11.16 s (*P* = 0.46).

**TABLE 2:** Ocular surface analyzer comparison previous and after both eyedrop 6-weeks treatment.

Variable		Baseline (*n* = 26)	After 6-weeks (*n* = 24)	*P-*value
0.40% HA + 0.20% GX	Conjunctival redness (Efron Scale)	2.35 ± 0.89 (0.00–4.00)	1.00 ± 0.56 (0.00–2.00)	<0.01
	Lipid layer thickness (Guillon Pattern)	0.96 ± 1.14 (0.00–4.00)	0.85 ± 0.92 (0.00–3.00)	0.60
	Tear meniscus height (Millimeters)	0.13 ± 0.05 (0.04–0.23)	0.14 ± 0.06 (0.00–0.31)	0.04
	First NIBUT (seconds)	5.07 ± 1.45 (3.80–9.44)	5.65 ± 2.29 (3.64–14.44)	0.49
	Mean NIBUT (seconds)	11.43 ± 6.09 (4.98–27.70)	12.54 ± 7.44 (5.32–38.20)	<0.01
	BUT (seconds)	8.69 ± 4.75 (2.00–21.00)	11.35 ± 4.71 (5.00–23.00)	<0.01
	OSDI (score points)	11.92 ± 8.09 (1.00–23.00)	8.15 ± 4.99 (0.00–17.00)	0.15
	SPEED (score points)	8.62 ± 3.76 (2.00–14.00)	7.38 ± 2.06 (2.00–10.00)	0.04
0.40% HA	Conjunctival redness (Efron Scale)	1.71 ± 0.80 (0.00–3.00)	0.79 ± 0.50 (0.00–2.00)	0.07
	Lipid layer thickness (Guillon Pattern)	0.71 ± 0.62 (0.00–2.00)	0.58 ± 0.65 (0.00–2.00)	0.65
	Tear meniscus height (Millimeters)	0.14 ± 0.03 (0.06–0.20)	0.14 ± 0.02 (0.10–0.18)	0.55
	First NIBUT (seconds)	5.87 ± 1.24 (3.92–7.88)	5.23 ± 1.14 (3.64–8.04)	0.41
	Mean NIBUT (seconds)	16.14 ± 9.69 (7.18–46.86)	14.32 ± 8.01 (5.74–33.74)	0.31
	BUT (seconds)	15.67 ± 11.75 (3.00–25.00)	11.04 ± 5.66 (4.00–25.00)	0.29
	OSDI (score points)	18.58 ± 7.24 (10.00–32.00)	11.92 ± 6.65 (4.00–25.00)	<0.01
	SPEED (score points)	11.08 ± 3.94 (5.00–18.00)	8.08 ± 3.87 (4.00–18.00)	<0.01

HA, Hyaluronic Acid; GX, Galacto Xyloglucan; NIBUT, Non-Invasive Break-Up Time; BUT, Break Up Time; OSDI, Ocular Surface Disease Index; SPEED, Standard Patient Evaluation Eye Disease.

### Break up time and subjective questionnaires

**FIGURE 2:**
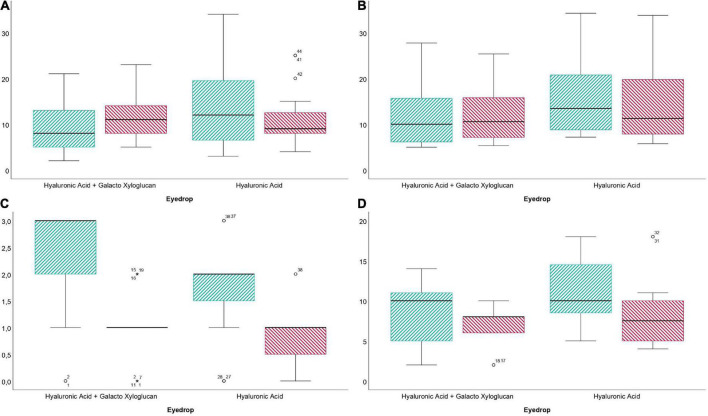
Box and plot graph differences before and after treatment with 0.40% hyaluronic acid with 0.20% galacto-xyloglucan (HA-GX) and 0.40% hyaluronic acid (HA). **(A)** Break up time differences before (striped, green) and after (striped, red) eyedrop treatment. **(B)** Mean non-invasive break-up time (NIBUT) before (striped, green) and after (striped, red) eyedrop treatment. **(C)** Conjunctival redness before (striped, green) and after (striped, red) eyedrop treatment and **(D)**. Standard patient evaluation eye disease (SPEED) before (striped, green) and after (striped, red) eyedrop treatment.

BUT test differences before and after eyedrop treatment are presented in Table 2 for participants in the HA-GX and HA-alone groups. Regarding differences between both groups, for the participants in the 0.40% HA-GX group, the changes between the previous and posterior BUT reported an increase of 2.65 ± 3.85 s, and participants in the HA-alone group achieved a decrease of 3.17 ± 9.69 s (*P* = 0.59). OSDI and SPEED differences before and after treatment are presented in Table 2 for participants in the HA-GX group and HA-alone group. Regarding differences between both groups, for participants in the 0.40% HA 0.20% GX group, a decrease of 3.76 ± 8.15 score points was reported between the previous and posterior OSDI questionnaire. Concerning the participants in the HA-alone group, the OSDI reported a decrease of 6.66 ± 6.59 score points was reported on the OSDI (*P* = 0.06). According to the results of the SPEED test, the participants in the HA-GX group achieved a decrease of 1.23 ± 3.49 points, and the HA-alone group achieved a decrease of 3.00 ± 3.65 points (*P* = 0.93). A statistically significant difference before and after eyedrop treatment box and blot graph is presented in Figure 2.

## Discussion

The results of our research show an improvement in both patient-reported symptomatology and tear break-up time in subjects treated with oral isotretinoin after using artificial tears composed of 0.4% HA and GX at 0.2%. The heterogeneous nature of DED and the variability of signs and symptoms do not allow an accurate diagnosis. There is evidence of the influence of the drug at the ocular level, negatively affecting the meibomian glands and goblet cells and, as a consequence, altering the lipid and mucin layers of the tear (7, 8) and aggravating the symptoms of dry eye (9). LBR is a consequence of dilation of the conjunctival blood vessels (29). The causes are quite varied and include meibomian gland dysfunction (30, 31). This clinical sign should be included in the diagnosis of DED (32).

Our results showed a statistically significant decrease in bulbar redness scores after 6 weeks of treatment in participants in the HA-GX group (2.35 ± 0.89–1.00 ± 0.56 degrees on the Efron Scale) (*P* < 0.01). Molina-Solana et al. (25) showed similar results. The authors described a significant decrease in LBR in a group of dry eye patients after 1 month of treatment with artificial tears containing HA-GX. HA has an effective moisturizing effect due to its high capacity to retain water (15, 16). On the other hand, the molecular structure of GX gives the polymer properties that mimic the natural mucosal barrier (23). The joint formulation of both molecules provides artificial tears with mucoadhesive properties and greater viscosity that, in the long term, increase hydration and reduce inflammation related to friction, conjunctival vasodilation and, as a consequence, LBR.

The lipid phase is an extremely thin tear film between the aqueous phase and the air, present in the anterior layer of the tear film. Its function is to delay the evaporation of the aqueous phase, and because the lipid surface tension exerts pressure on the aqueous phase, it keeps the aqueous phase from spilling (33). Alterations in the quantity, quality and composition of the lipid phase are related to DED (34, 35). Furthermore, tear meniscus height (TMH) is related to total tear volume and tear secretion rate. Measurement of both lipid layer thickness (LLT) and TMH is valuable for diagnosing DED (36).

Oral administration of isotretinoin alters the function and structure of the meibomian gland and inhibits lipid production, generating rapid tear evaporation (7). It is to be expected that the participants included in the research demonstrate alterations in the function and structure of the meibomian glands as a result of isotretinoin. Recently, artificial tears that include lipids in their composition have been considered important, since they add thickness to the lipid layer of the tear film, reducing evaporation (37–40). An artificial tear containing lipids is capable of increasing the thickness of the lipid layer within 15 min of instillation (38). The formulation of the eye drops in our study did not contain lipids, and this situation could justify the fact that neither LLT nor TMH improved after 6 weeks of treatment.

The innermost layer of the tear film is a thin layer of mucin produced almost entirely by the goblet cells of the conjunctiva. It spreads over the surface of the corneal epithelium and the conjunctiva, making them hydrophilic and allowing them to be highly hydrated and lubricated (41–43). Isotretinoin alters the conjunctival epithelium, affecting the morphology, and density of goblet cells and interfering with mucin production (8, 44). In the evaluation of the ocular surface, we include the non-invasive measurement with OSA of two tear breakup times, the first breakup time (FNIBUT) and the mean breakup time (MNIBUT), which is the average of all the tear film breakups that occur throughout the cornea. In a longitudinal approach, the results showed an increase in MNIBUT after 6 weeks of treatment in participants in the HA-GX group (11.43 ± 6.09 s – 12.54 ± 7.44 s) (*P* < 0.01). We also measured BUT invasively with fluorescein. The results showed a statistically significant increase in participants in the HA-GX group (8.69 ± 4.75 s – 11.35 ± 4.71 s) (*P* < 0.01). There were similarities between the structure of GX, tamarind seed polysaccharide (TSP) and the mucin MUC1 present in the epithelium of the cornea and conjunctiva (24, 45), which could be the reason for the increase in the tear breakup time that we observed. The HA-GX combination generates a synergistic action on the anterior surface, and several authors have confirmed its efficacy in ED treatment (25–27, 46).

In addition, we used two questionnaires, the OSDI (score) and SPEED (score), to classify the degree of dry eye according to its symptoms (47). In a longitudinal approach, our results showed a statistically significant decrease after 6 weeks of treatment in the SPEED scores in participants in the HA-GX group, while participants in the HA group showed a significant decrease in the scores of both questionnaires, OSDI and SPEED. The GX in a molecule similar to mucin (23) provides viscosity to the tear. After its application, it can cause blurred vision and discomfort related to the texture of the product, which makes it difficult to spread evenly over the ocular surface. This could be the reason that justifies the fact that participants in the HA-GX group did not report improvement in symptoms when evaluated with the OSDI.

### Future research lines and limitations

Regarding strengths and limitations, to the best of our knowledge, this clinical study demonstrated the efficacy of two types of eyedrops in oral isotretinoin for acne vulgaris for the first time. In addition, non-invasive ocular surface analyzer measurements were used. Within the limitations, the sample size and follow-up of the research could be improved to confirm these results. Furthermore, a double-blind design should reduce patient bias. Future lines of research should include the isotretinoin dose analysis and correlate it with the ocular surface signs and symptoms.

Within the future research lines, eyedrop manufacturer laboratories should open the option of producing personalized lubricants for each patient. Regarding dry eye disease pathophysiology, one or more tear layers will be affected, so a different excipient in the eyedrop composition is needed. Therefore, the indication for dry eye disease treatment should include a thorough dry eye examination and evaluation of the causes that instigated it.

## Conclusion

In conclusion, hyaluronic acid in combination with galacto-xyloglucan significantly decreased limbal and bulbar conjunctival redness and SPEED subjective dry eye disease symptoms. Galacto-xyloglucan also increased the BUT and mean NIBUT compared to hyaluronic acid alone.

## Data availability statement

The data presented in this study are available on request from the corresponding author. The data are not publicly available due to their containing information that could compromise the privacy of research participants.

## Ethics statement

The studies involving human participants were reviewed and approved by the Ethical Committee Board of the University of Seville. The patients/participants provided their written informed consent to participate in this study.

## Author contributions

MS-G, CD-H-C, and J-MS-G: conceptualization and methodology. MS-G, CD-H-C, CM-L, and J-MS-G: writing—original draft preparation, writing—review and editing, and supervision. All authors have read and agreed to the published version of the manuscript.

## Conflict of interest

The authors declare that the research was conducted in the absence of any commercial or financial relationships that could be construed as a potential conflict of interest.

## Publisher’s note

All claims expressed in this article are solely those of the authors and do not necessarily represent those of their affiliated organizations, or those of the publisher, the editors and the reviewers. Any product that may be evaluated in this article, or claim that may be made by its manufacturer, is not guaranteed or endorsed by the publisher.
